# Effects of 3,4-diaminopyridine on myasthenia gravis: Preliminary results of an open-label study

**DOI:** 10.3389/fphar.2022.982434

**Published:** 2022-08-16

**Authors:** Marco Ceccanti, Laura Libonati, Gabriele Ruffolo, Pierangelo Cifelli, Federica Moret, Vittorio Frasca, Eleonora Palma, Maurizio Inghilleri, Chiara Cambieri

**Affiliations:** ^1^ Neuromuscular Disorders Unit, Department of Human Neurosciences, Sapienza University, Rome, Italy; ^2^ Department of Physiology and Pharmacology, Institute Pasteur- Fondazione Cenci Bolognetti, University of Rome Sapienza, Rome, Italy; ^3^ IRCCS San Raffaele Pisana, Rome, Italy; ^4^ Department of Applied Clinical and Biotechnological Sciences, University of L'Aquila, L'Aquila, Italy

**Keywords:** myasthenia gravis, acetylcholine receptors, repetitive nerve stimulation, quantitative myasthenia gravis test, anti-AChR antibodies, 3,4-diaminopyridine (3,4-DAP)

## Abstract

**Background:** 3,4-diaminopyridine (3,4-DAP) can lead to clinical and electrophysiological improvement in myasthenic syndrome; it may thus represent a valuable therapeutic option for patients intolerant to pyridostigmine.

**Objective:** to assess 3,4-diaminopyridine (3,4-DAP) effects and tolerability in patients with anti-AChR myasthenia gravis.

**Method:** Effects were monitored electrophysiologically by repetitive nerve stimulation (RNS) and by standardized clinical testing (QMG score) before and after a single dose administration of 3,4-DAP 10 mg per os in 15 patients. Patients were divided according to their Myasthenia Gravis Foundation of America (MGFA) class into mild and severe.

**Results:** No significant side effects were found, apart from transient paresthesia. 3,4-DAP had a significant effect on the QMG score (*p* = 0.0251), on repetitive nerve stimulation (*p* = 0.0251), and on the forced vital capacity (*p* = 0.03), thus indicating that it may reduce the level of disability and the decremental muscle response. When the patients were divided according to the MGFA classification, 3,4-DAP showed a positive effect in the *severe* group, either for the QMG score (*p* = 0.031) or for the RNS decrement (*p* = 0.031). No significant difference was observed in any of the outcome measures within the *mild* group (*p* > 0.05). A direct effect of 3,4-DAP on nicotinic ACh receptors (nAChRs) was excluded since human nAChRs reconstituted in an expression system, which were not affected by 3,4-DAP application.

**Conclusion:** Our results suggest that 3,4-DAP may be a useful add-on therapy, especially in most severe patients or when immunosuppressive treatment has not yet reached its full effect or when significant side-effects are associated with anticholinesterase.

## Introduction

Myasthenia gravis (MG) is a relatively rare acquired disorder of the neuromuscular junction (NMJ), mediated by autoantibodies directed towards postsynaptic proteins. Patients present with typically fluctuating muscle weakness—worsening with exertion—most commonly involving extrinsic ocular muscles. In most patients, IgG1 and IgG3 to acetylcholine receptors (AChR) can be detected and cause loss of AChR at the NMJ through a complement-mediated damage of the postsynaptic membrane. Moreover, these antibodies can accelerate AChR internalization and degradation and occasionally directly inhibit AChR signaling (Vincent, 2020).

About 5–8% of patients present IgG4 to muscle-specific-tyrosine-kinase (MuSK), a protein involved in AChR clustering and in the maintenance of the postsynaptic membrane. IgG4 anti-MuSK cannot activate complement and exert their effect by directly blocking the MuSK. These patients usually show a different and more severe clinical pattern, with more prominent facial and bulbar muscle weakness and atrophy; those patients are often unresponsive to anticholinesterase and may have increased sensitivity to ACh(Hatanaka et al., 2005).

The current treatment of MG mainly relies on drugs that nonspecifically suppress the immune system, thus reducing the level of antibodies (Gilhus et al., 2019). Among symptomatic therapies, anticholinesterases enhance the bioavailability of ACh at the synaptic cleft; pyridostigmine bromide represents a first-line medication in patients with anti-AChR MG. Nevertheless, over long periods, only a few patients show an optimal disease control; furthermore, adverse effects of anticholinesterases—mainly resulting from the stimulation of muscarinic AChR in the autonomic nervous system—are common and often require dose adjustments.

Aminopyridines—such as 4-aminopyridine (4-AP) and 3,4-diaminopyridine (3,4-DAP) are able to block voltage-gated potassium channels (VGKCs), thereby prolonging the depolarization of nerve action potential. This increases the opening time of voltage-gated calcium channels (VGCCs), thus increasing presynaptic calcium entry which enhances ACh release. 3,4-DAP has a lower brain penetration than 4-AP, showing weaker effects on central nervous system (Lemeignan et al., 1984; Flet et al., 2010). It is a safe and effective treatment in Lambert-Eaton myasthenic syndrome (LEMS), a rare autoimmune disorder characterized by a reduced release of ACh by the presynaptic terminal of the NMJ as a consequence of autoantibodies which target the pre-synaptic VGCCs(Sanders et al., 2018, 4).

Previous studies showed that 4-AP and 3,4-DAP lead to clinical and electrophysiological improvement in a very small cohort of patients with MG and may thus be valuable supplementary treatments in the disease (Lundh et al., 1979, 1985).

3,4-DAP is well tolerated and effective in improving neuromuscular transmission also in MuSK MG (Evoli et al., 2016; Bonanno et al., 2018); in this form of disease, a cholinergic hypersensitivity is often present due to a relative acetylcholinesterase (AChE) deficiency—3,4-DAP may represent a valuable therapeutic option for patients intolerant to pyridostigmine.

Congenital myasthenic syndromes (CMS) are the rarest of myasthenic disorders, caused by mutations affecting crucial presynaptic, synaptic, or postsynaptic proteins involved in neuromuscular transmission. 3,4-DAP has been shown to be a useful treatment option in CMS(Verma et al., 2016).

3,4-DAP reverses respiratory depression and neuromuscular weakness in murine models of acute and chronic botulism (Vazquez-Cintron et al., 2020). The medication seems to increase the quantal content and promote neurotransmission in botulinum-intoxicated nerve terminals through two functionally distinct mechanisms: by increasing the probability of neurotransmission at non-intoxicated release sites and by eliciting persistent production of toxin-resistant endplate potentials from nerve terminals (Bradford et al., 2018).

The primary objective of this study was to evaluate the effects of 3,4-DAP 10 mg (a dose reported to be effective in a placebo-controlled, double-dummy, double-blind, randomized, crossover study in nine patients with LEMS(Wirtz et al., 2009)) in patients with AChR-MG, as tested by the quantitative myasthenia gravis (QMG) score, the measurement of the forced vital capacity (FVC) using the spirometry and a motor nerve conduction study (NCS) with low frequency repetitive nerve stimulation (RNS). Secondary objectives included the tolerability of the medication in patients enrolled in this study, and to evaluate any possible correlation between the variations observed and the clinical severity.

Furthermore, we used frog’s oocytes (from *Xenopus laevis*) to microtrasplant human muscle nAChRs to test whether 3,4-DAP could directly influence the ACh currents and desensitization.

## Methods

This is a single center interventional study conducted in the Neuromuscular Disorders Unit of the Policlinico Umberto I, Sapienza University of Rome, Italy. The study was approved by the institutional review board and performed in accordance with the Declaration of Helsinki. All patients provided written informed consent before inclusion in the study. Patients were consecutively recruited at the clinic for rare neuromuscular diseases between January and May 2021.

### Subjects

We included patients aged 18 years and older, with a diagnosis of anti-AChR antibody-positive myasthenia gravis as measured using an Enzyme-linked immunosorbent assays (RSR Limited, Acetylcholine Receptor Autoantibody ELISA Kit, sensitivity 92,0%, specificity 99,8%), in treatment with AChE inhibitors and immunosuppressors at a stable dose for at least 6 months. Exclusion criteria included history of epilepsy or asthma, the presence of a prolonged QTc interval on the electrocardiogram, pregnancy or breast-feeding, and positivity to antibodies to VGCC or MuSK.

All patients were evaluated at baseline T0 (immediately before taking 3,4-DAP, 10 mg per os and approximately 12 h after the last intake of pyridostigmine at their usual dose) and at T1 (60 min after the intake of 3,4-DAP 10 mg per os). At each timepoint, all patients underwent a clinical neurological examination including the QMG score, a commonly used outcome measure in MG, which also includes the measurement of the FVC at spirometry; patients also underwent motor NCS with low frequency RNS from axillary and masseter nerves.

Patients remained under medical observation for 5 h after the intake of 3,4-DAP 10 mg; they were asked to report any side effect and vital signs were measured and an electrocardiogram was performed approximately 1 hour after the medication intake.

#### QMG score

The QMG scoring system represents an objective clinical evaluation tool in MG (Bedlack et al., 2005). It is based on quantitative testing of sentinel muscle groups usually affected by the disease and consists of 13 items measuring endurance or fatigability, taking thus into account the characteristic fluctuating nature of the symptoms. Each item is scored from 0 to 3, where 3 represents a severe impairment. The total QMG score ranges from 0 to 39, with higher scores associated with greater disease severity. The items studied in QMG scores are as follows: ptosis, diplopia, orbicularis oculi, weakness, swallowing a cup of water, speech, percent predicted FVC at spirometry, bilateral grip strength, bilateral arm endurance, bilateral leg endurance, and neck flexion endurance.

The QMG score was performed by the same neurologist at each timepoint.

#### Electrophysiological study

Motor NCS was performed using a Micromed Myoquick 1400 EMG machine (Micromed S.p.A., Treviso, Italy); Ag/AgCl surface electrodes were applied according to a conventional belly-to-tendon method. Filter settings were 5 Hz—5 kHz, sweep duration was 30 ms, and sensitivity was 1 mV/division.

The 3 Hz RNS was performed by supramaximally stimulating the masseteric nerve (Pavesi et al., 2001), a branch of the trigeminal nerve, between the mandibular notch and the zygomatic arch and the axillary nerve at the Erb point. The stimulating dipole consisted of a monopolar needle with a 0.40 mm diameter that acted as the cathode and a surface Ag-AgCl electrode that acted as the anode. We measured the decremental response, as the percentage change of the compound motor action potential (CMAP) amplitude between the fourth and the first electrical stimulus.

To ensure a better reproducibility of the electrophysiological data, surface electrodes were not removed between T0 and T1 and the stimulation sites were marked with a pen prior to removing the needle electrode. The technician who performed the study was not blinded to the patient’s status (T0 vs. T1 assessment).

In each patient, the nerve showing the greatest CMAP decrement at T0 was selected for the statistical analysis.

#### Spirometry

The spirometry was performed using a Winspiro PRO 5.8 spirometer. Percent predicted FVC was measured. The best value recorded out of three consecutive trials was registered.

#### Microtrasplantation in oocytes and voltage-clamp recordings

Concerning voltage-clamp recordings in *Xenopus laevis* oocytes, we transplanted membranes obtained from muscle biopsy (anterior tibialis) of two control patients (65 and 40 years old, hospitalized for polytrauma from 1 to 2 months; see Palma et al., 2011) (Palma et al., 2011). This simple approach permits to study nAChRs extracted from human muscle biopsies conserving their native characteristics (Palma et al., 2011). The muscle specimens (about 5 mg) were frozen in liquid nitrogen immediately after biopsy and stored at −80°C until use. Membranes were prepared as previously described (Palma et al., 2002). Preparation of *Xenopus laevis* oocytes, cytoplasmic injection procedures, and intracellular voltage-clamp recordings were performed as described elsewhere (Palma et al., 2002). Desensitization of ACh evoked currents (I_ACh_) during repetitive ACh applications (200 μM for 7 s at 60-s intervals) was quantified by expressing the peak amplitude of the sixth response as a percent of the peak amplitude of the first response.

The use of female *Xenopus laevis* frogs was conformed to the institutional policies and guidelines of the Italian Ministry of Health (authorization no. 427/2020-PR). The oocyte Ringer solution (OR) and acetylcholine (ACh) were dissolved as previously described (Musarò et al., 2019). The 3,4 diaminopyridine (3,4 DAP) was purchased from Sigma-Aldrich and dissolved in sterile water according to the manufacturer instructions.

### Statistical analysis

The SPSS 25.0 program was used for the statistical analysis of the clinical data.

Because of the small sample size and in consideration of the not Gaussian distribution of the data, the Wilcoxon signed-rank test was used to compare the QMG score, the FVC, and the decrement of the RNS at the time points. The same test was performed after dividing the patients according to their MGFA classification.

*p* values < 0.05 were considered as statistically significant. Clinical and electrophysiological data are expressed as mean ± standard deviation (SD).

Concerning the voltage-clamp recordings in oocytes before statistical analysis, normal distribution was assessed with Shapiro-Wilk Test and parametric (*Paired t-test*) or nonparametric (*Wilcoxon Signed Rank Test*) analyses were used accordingly. Those data are expressed as mean ± SEM. Sigmaplot 12 software (Systat Software Inc.; San Jose, CA, U.S.A.) was used for statistical analysis of *Xenopus* oocytes electrophysiological recordings data.

## Results

We included 15 patients in the study (9 males, 6 females). The mean age at the time of the study was 58,47 ± 10,97, ranging from 36 to 71 years.

**FIGURE 1:**
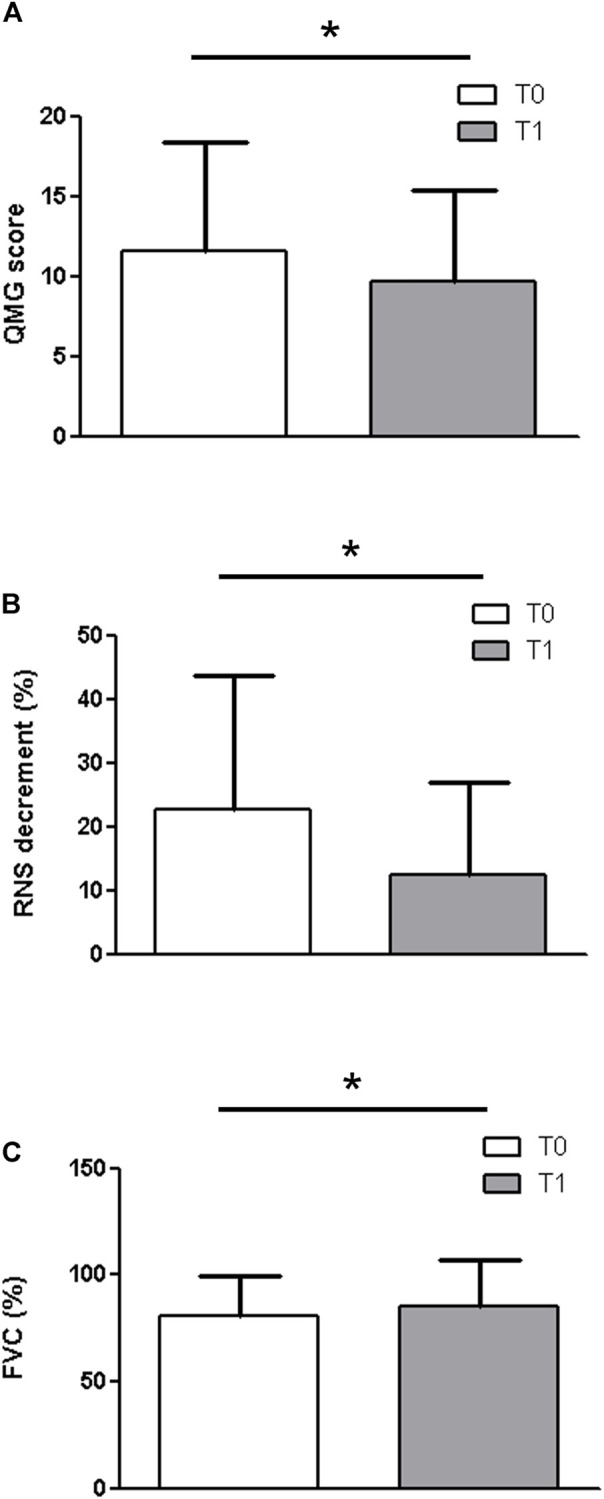
When considering the entire group of patients, a significant difference was observed at T1 for the QMG score **(A)** (T0: 11,6 ± 6.80; T1: 9.67 ± 5.7 *p* = 0.0251), the RNS decrement **(B)** (T0: 22.73 ± 21.06; T1: 12.53 ± 14.28. *p* = 0.0251) and for the FVC **(C)** (T0: 80.53 ± 18.75; T1: 85.47 ± 21.44. *p* = 0. 03).

**FIGURE 2:**
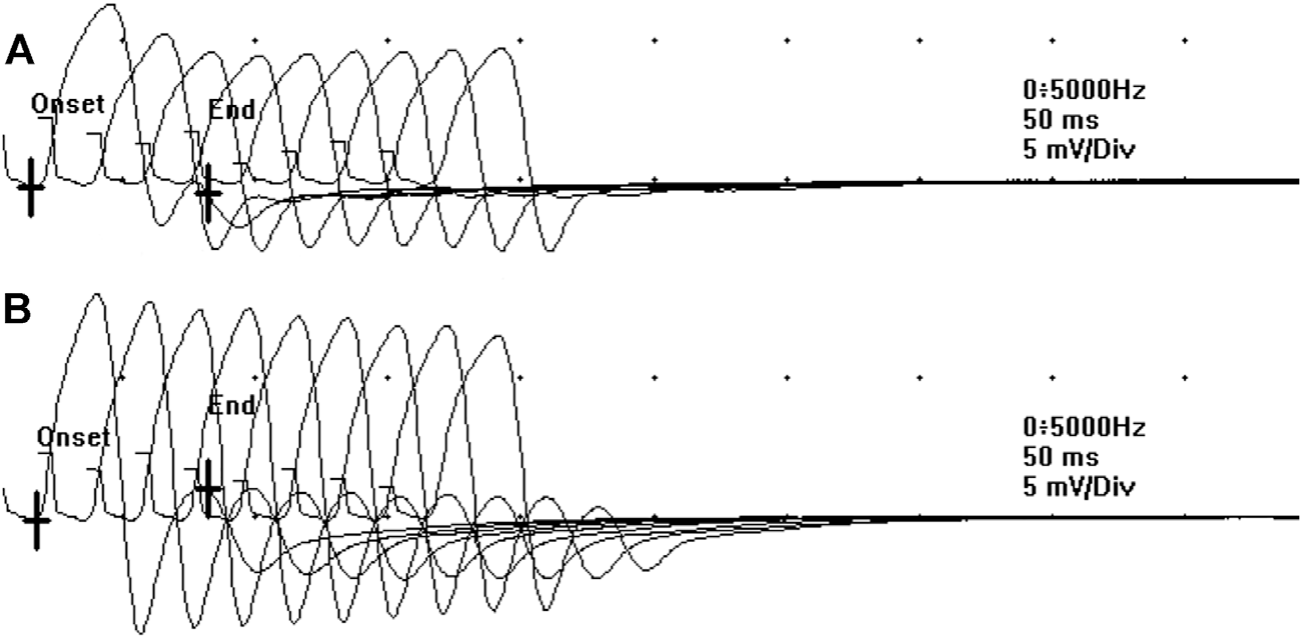
The decremental response observed at the RNS at T0 **(A)** significantly ameliorated after the administration of 3,4-DAP 10 mg per os, at T1 **(B)**.

When considering the entire group of patients (Figure 1), a significant difference was observed at T1 for the QMG score (T0: 11,6 ± 6.80; T1: 9.67 ± 5.7 *p* = 0.0251), the RNS decrement (T0: 22.73 ± 21.06; T1: 12.53 ± 14.28. *p* = 0.0251) (Figure 2) and for the FVC (T0: 80.53 ± 18.75; T1: 85.47 ± 21.44. *p* = 0. 03).

**FIGURE 3:**
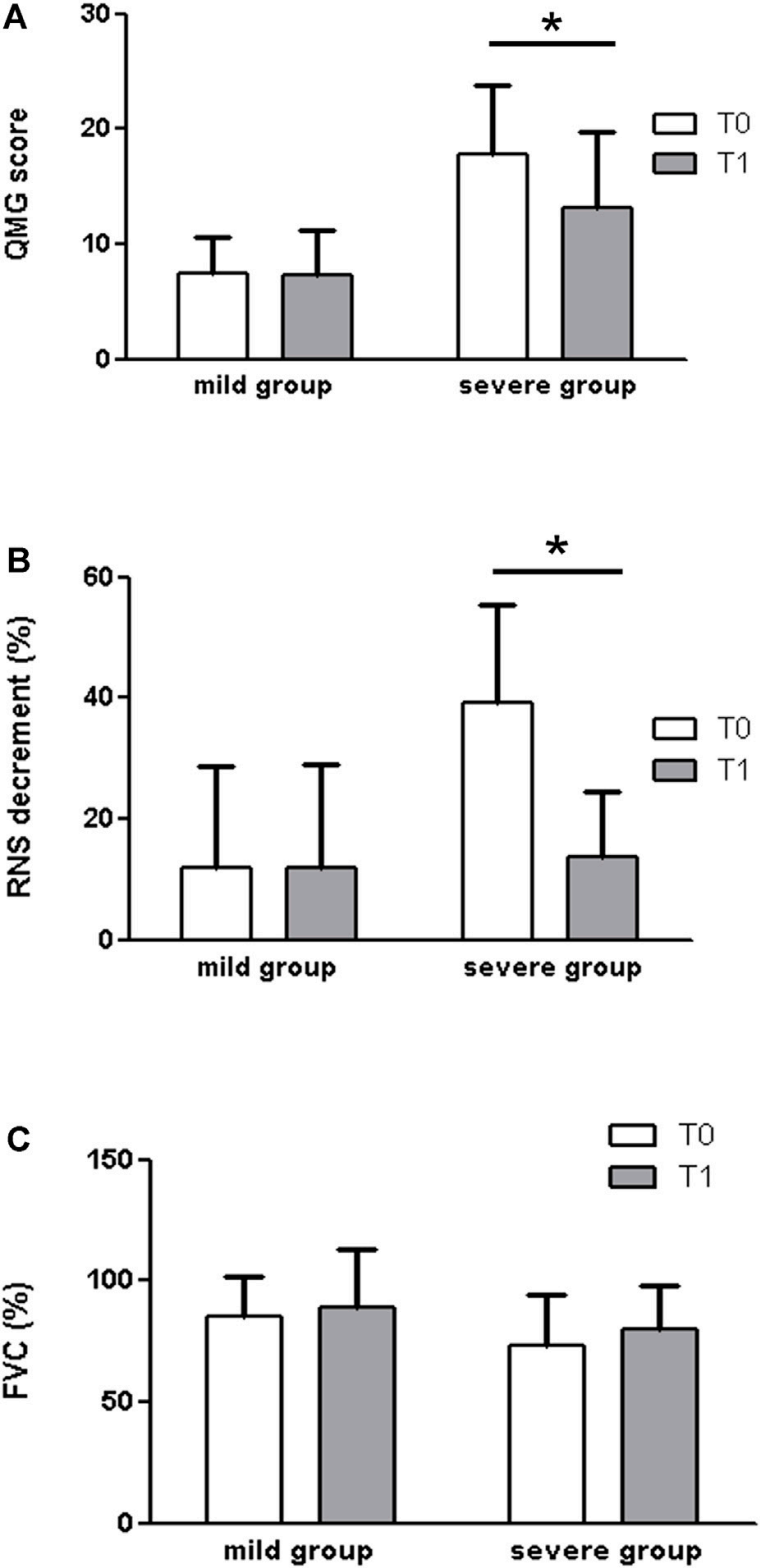
When we divided patients by grouping milder (MGFA classes IIa and IIb, *n* = 9) and more severe (MGFA classes III-IV, *n* = 6) forms of disease, what emerged is that 3,4-DAP showed a positive effect in the severe group, by measures of the QMG score **(A)** (T0: 17.83 ± 6.01; T1: 13.17 ± 6.58. *p* = 0.031), the RNS decrement **(B)** (T0: 39.25 ± 16.25; T1: 13.67 ± 10.71. *p* = 0.031), and a trend towards significance for the FVC **(C)**(T0: 73 ± 21.14; T1: 80 ± 17.83 *p* = 0.063). No significant difference was observed in any of the outcome measures within the mild group (*p* > 0.05).

When we divided patients by grouping milder (MGFA classes IIa and IIb, *n* = 9) and more severe (MGFA classes III-IV, *n* = 6) forms of the disease (Figure 3), what emerged is that 3,4-DAP showed a positive effect in the *severe* group, either for the QMG score (T0: 17.83 ± 6.01; T1: 13.17 ± 6.58. *p* = 0.031) or for the RNS decrement (T0: 39.25 ± 16.25; T1: 13.67 ± 10.71. *p* = 0.031) and a trend towards significance for the FVC (T0: 73 ± 21.14; T1: 80 ± 17.83 *p* = 0.063). No significant difference was observed in any of the outcome measures within the *mild* group (*p* > 0.05).

**FIGURE 4:**
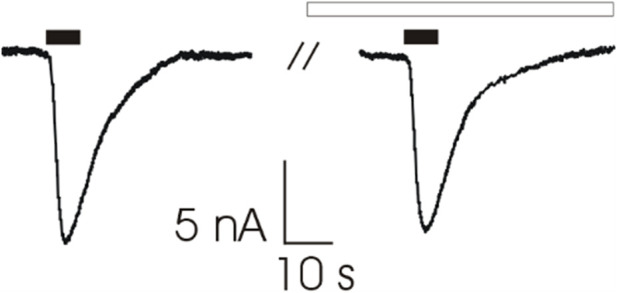
Representative traces of ACh-evoked currents (200 μM, 7s, black bars) recorded from *Xenopus* oocytes microtransplanted with muscle tissue (tibialis anterior muscle; 2 individuals) before (Left) and after (Right) 120 s of pre-incubation with 3,4-DAP (100 ng/ml, white bar). Holding potential (V_H_) was -60 mV. Note that 3,4-DAP did not affect ACh-evoked current’s amplitude (13.1 ± 2.6 nA *versus* 12.2 ± 2.3 before and after pre-incubation respectively, n = 12; *p* = 0.256, Wilcoxon signed rank test).

To verify that patients’ clinical improvement was not due to a direct modulation of AChR function by 3,4 DAP, we microtransplanted human muscle membrane preparation from two control patients (tibialis anterior) into *Xenopus* oocytes for recording ACh evoked current response (I_ACh_) with or without preincubation with 3,4 DAP (120 s, 100 ng/ml). We found that the I_ACh_ amplitude was not modified in 12 cells (13.1 ± 2.6 nA *versus* 12.2 ± 2.3; *p* = 0.256) (Figure 4). In addition, no significant difference was found as current desensitization after the 3,4 DAP preincubation (n = 8; 54.0 ± 4.9% *versus* 60.0 ± 3.4%; *p* = 0.125). Altogether, these results exclude a direct effect of 3,4 DAP on AChRs at the NMJ.

## Discussion

This is a single-arm open-label study investigating the effects of 3,4-DAP 10 mg in a small group of AChR-MG patients. Our results provide evidence that 3,4-DAP can be effective in MG, which was already previously demonstrated (Lundh et al., 1979, 1985), and showed a major effect in mostly severely affected patients. Furthermore, we demonstrated that patients’ clinical improvement was not due to a direct modulation of AChR currents and desensitization by 3,4 DAP by microtrasplanting human muscle nAChRs into frog’s oocytes from *Xenopus laevis*.

Overall, 3,4-DAP was well-tolerated; no patient showed major side effects, 9 patients (60%) reported transient perioral paresthesia and tingling in the mouth or in the fingers and toes.

Our data show a clinical and electrophysiological effect of 3,4-DAP in AChR-MG, as demonstrated by a significant reduction of the QMG score, FVC value, and of the decrement of the RNS in the whole sample of patients; this result is in line with previous studies (Lundh et al., 1979, 1985). Our patients were tested in the absence of pyridostigmine, and after a single administration of 3,4-DAP 10 mg; nevertheless, we know from previous works that the combination of the two drugs results in a greater response than that observed with pyridostigmine alone, and that the prolonged and combined therapy of the two treatments for several weeks determines a lasting response time (Lundh et al., 1979, 1985).

To verify if there were any significant differences of the effect of 3,4-DAP according to the severity of the clinical status, we divided patients by grouping milder (MGFA classes IIa and IIb) and more severe (MGFA classes III-IV) forms of the disease; what emerged is that no significant difference was observed in any of the outcome measures within the *mild* group; notably, measures of QMG score and RNS fall within normality range in milder forms of disease, which are included in this group, and any difference in these measures would then be difficult to be detected in our cohort. Conversely, 3,4-DAP showed a significant positive effect in the *severe* group, either for the QMG score (improvement at T1 of 4,66 points, which is considered as clinically significant) or for the RNS decrement and a trend towards significance for the FVC. This difference observed between the severe and mild group could be simply due to the low number of patients in each group and the greater QMG/RNS difference in the severe group, which may have resulted in a statistically significant difference even with a low sample size.

A homeostatic mechanism seems to gradually adjust the presynaptic neurotransmitter release to the actual level of postsynaptic sensitivity for the neurotransmitter ACh. In this way, reliable transmission remains guaranteed (Plomp, 2017). In MG, this homeostatic mechanism—even if maximally active - no longer suffices to counteract the postsynaptic changes and muscle weakness results.

Our results in the *severe* group seem to suggest that in those patients the homeostatic mechanisms are enhanced, but not maximally active, thus allowing a further increase of ACh release at the presynaptic terminal after 3,4-DAP administration.

AChRs are also present at the presynaptic site of the NMJ, and they are of two types, ionotropic (nAChR) and metabotropic (muscarinic, mAChRs) (Garcia et al., 2005). It was shown that inhibition of both AChE and butyrylcholinesterase (BChE)—a closely related enzyme anchored at the terminal Schwann cell (TSC) that can hydrolyse ACh and whose physiological relevance is not fully understood—decreases the probability of ACh release regardless the type of mAChRs (Minic et al., 2003). nAChRs can be localized at the TSC and act as a sensor for spillover of ACh adjusted by BChE. The use of nonselective AChEs inhibitors does not only potentiate the effect of ACh on postsynaptic nAChRs, but also causes additional activation of presynaptic autoregulatory pathways, ultimately leading to a reduction of ACh quanta release (Wessler, 1989; Petrov et al., 2018). This could contribute to explain why the *severe* group of patients in our study showed a better response to 3,4-DAP; in this subgroup of patients, higher doses of pyridostigmine were used (Table 1), and in consideration of the low selectivity of pyridostigmine for AChE versus BChE we can hypothesize a relative reduction of quantal transmitter release and thus a consequent better response to 3,4-DAP (Petrov et al., 2014; Soukup et al., 2017).

**TABLE 1:** **–** daily total posology of pyridostigmine and immunosuppressant therapy of the patients enrolled in the study.

Patient #	MGFA class	Pyridostigmine therapy	Immunosuppressant
1	II	90 mg/day	Azathioprine 50 mg/day
2	II	90 mg/day	Prednisone 12,5 mg/day
3	II	90 mg/day	Azathioprine 50 mg/day Prednisone 10 mg/day
4	II	30 mg/day	Azathioprine 100 mg/day Prednisone 10 mg/die
5	II	60 mg/day	Azathioprine 100 mg/day Prednisone 15 mg/die
6	II	90 mg/day	Azathioprine 100 mg/day Prednisone 25 mg/die
7	II	120 mg/day	Azathioprine 100 mg/day Prednisone 10 mg/die
8	II	30 mg/day	Azathioprine 50 mg/day
9	II	120 mg/day	Azathioprine 50 mg/day
10	III	120 mg/day	Azathioprine 100 mg/day
11	III	120 mg/day	Azathioprine 100 mg/day Prednisone 10 mg/die
12	III	120 mg/day	Azathioprine 100 mg/day
13	III	180 mg/day	Prednisone 25 mg/die
14	IV	270 mg/day	Azathioprine 100 mg/day Prednisone 25 mg/die
15	IV	150 mg/day	Azathioprine 100 mg/day

MGFA, myasthenia gravis foundation of america.

Our data on human AChRs suggest that a direct action of 3,4-DAP on the AChRs can be excluded because in oocytes transplanted with human muscle membranes, we did not record any difference in ACh current amplitude nor in current desensitization. An effect of 3,4 –DAP on VGKC at the postsynaptic level of NMJ is also unlikely, because these channels are poorly expressed in the junctional area and limited to Kv 7.4 and Kv 7.5 that are not sensitive to the block by 4-AP (Robbins, 2001; Khammy et al., 2018). Therefore, we could hypothesize that the drug can exert its effects by a presynaptic blocking of the VGKCs, which increases the calcium entry, thus allowing the release of the neurotransmitter ACh. Unlike pyridostigmine, 3,4-DAP may determine a rapid and burst increase of ACh levels in the synaptic cleft. This would explain a synergistic nonoverlapping effect of 3,4-DAP and AChE inhibitors in patients with MG.

Our study has several limitations. First of all, the low number of patients enrolled could have led to not identifying statistically significant differences in the outcome measures, especially in the *mild* group. Furthermore, the absence of a blinding and the lack of a control group did not allow us to exclude a possible placebo effect, especially in the differences observed in the QMG test; nevertheless, a significant improvement in the decremental response at the RNS—which objectively investigates the neuromuscular safety factor—let us speculate about a true pharmacological effect of the 3,4-DAP rather than a placebo effect. Finally, our inclusion criteria did not set a minimum value for the QMG or for the decremental response at the RNS, thus allowing patients who presented clinical and electrophysiological measures within the normal limit to be enrolled in the study; this could have contributed to the lack of effects observed in the *mild* group. A well powered and appropriately designed, double-blinded, randomized-controlled clinical trial is required to better assess the efficacy of 3,4-DAP over time in AChR-MG patients; stricter inclusion criteria concerning the baseline QMG and RNS measures are necessary to better investigate the different subgroups of patients. Notwithstanding the aforementioned limitations, the preliminary exploratory results of our study support a potential benefit of 3,4-DAP use in AChR-MG.

## Conclusion

In conclusion, our exploratory study suggests a potential benefit of 3,4-DAP treatment in patients with anti-acetylcholine receptor antibody-positive myasthenia gravis and should stimulate further research into the role of this treatment in different severity forms of this disease and In the possibility of targeting specific muscular regions (as assessed by the QMG subscores).

Our study suggested that 3,4-DAP is well tolerated and determines a significant clinical and electrophysiological effect in more severely affected patients with AChR-MG patients, likely due to a different action than that exerted by AChE inhibitors. This medication could represent a valuable add-on therapy, especially while waiting for immunomodulatory or immunosuppressive therapy to take effect, or in those patients who show poor tolerance to AChE inhibitors because of side effects. Additionally, MuSK-MG patients could further benefit from therapy with 3,4-DAP, in consideration of the frequent lack of response to AChE inhibitors and of the absence of homeostatic mechanisms usually adopted in this group of patients. Finally, 3,4-DAP may be a useful treatment for disease acute worsening and clinical exacerbations.

## Data Availability

The raw data supporting the conclusions of this article will be made available by the authors, without undue reservation.

## Abbreviations

ACh, acetylcholine; AChE, acetylcholinesterase; AChR, acetylcholine receptor; AP, aminopyridine; BChE, butyrylcholinesterase; CMAP, compound motor action potential; CMS, congenital myasthenic syndrome; DAP, diaminopyridine; FVC, forced vital capacity; LEMS, Lambert-Eaton myasthenic syndrome; MG, myasthenia gravis; MGFA, myasthenia gravis foundation of America; MuSK, muscle specific kinase; NCS, nerve conduction study; NMJ, neuromuscular junction; OR, oocytes Ringer solution; QMG, quantitative myasthenia gravis; RNS, repetitive nerve stimulation; TSC, terminal Schwann cell; VGCC, voltage-gated calcium channel; VGKC, voltage-gated potassium channel.
